# Resting-State EEG Features of Cognitive Fluctuations Across the Lewy Body Disease Spectrum

**DOI:** 10.21203/rs.3.rs-9193762/v1

**Published:** 2026-03-31

**Authors:** Ahmed Negida, Sarah K. Lageman, Kenchiro Ono, Md Moinul Ahsan, Nitai Mukhopadhyay, Matthew J. Barrett

**Affiliations:** Virginia Commonwealth University; Virginia Commonwealth University; Virginia Commonwealth University; Virginia Commonwealth University; Virginia Commonwealth University; Virginia Commonwealth University

**Keywords:** Lewy body dementia, cognitive fluctuations, electroencephalography, dominant frequency, biomarker, Parkinson’s disease dementia

## Abstract

**Background:**

Cognitive fluctuations (CF) are a core feature of Lewy body dementia (LBD) that negatively impact quality of life. Despite their prevalence and negative impact, CF are challenging to identify clinically. The goal of this study was to identify easily obtainable EEG features associated with CF across the spectrum of Lewy body disease (LB).

**Methods:**

We conducted a cross-sectional study of 53 patients prospectively enrolled through the outpatient clinics of the Department of Neurology at Virginia Commonwealth University. Based on the Clinician Assessment of Fluctuations, participants were categorized as LB disease without CF (LB-CF) or LB disease with CF (LB + CF). All participants underwent a resting-state EEG recording with eyes closed for at least 3 minutes. EEG data were preprocessed and the following features were extracted: dominant frequency (DF), dominant frequency variability (DFV), dominant frequency prevalence (DFP), and individual alpha peak frequency (IAF). We applied Kruskal-Wallis tests and logistic regression models to evaluate differences in EEG features between study groups.

**Results:**

We analyzed EEG features for 29 LB + CF and 24 LB-CF participants. Compared to the LB-CF group, the LB + CF group had significantly lower DF, DFP-alpha, and IAF in all derivations (all *p* < 0.05). In logistic regression analysis adjusting for age, sex, and MoCA score, these EEG features remained significant predictors of group (LB + CF vs. LB-CF, all *q* < 0.05). The elastic-net logistic regression model identified five predictors (anterior DFP-alpha, anterior DFP-theta, anterior DF, temporal DF, temporal IAF, and posterior DFP-pre-alpha) achieving an AUC of 0.86, whereas anterior DFP-alpha alone achieved an AUC of 0.85.

**Conclusion:**

Among patients with LB disease, resting-state EEG features were associated with CF and were significant predictors of CF even after adjustment for age, sex, and MoCA. The development of an EEGbased biomarker of CF using this set of features could improve diagnosis of Lewy body dementia.

## Introduction

Lewy body dementia (LBD), which includes Parkinson’s disease dementia (PDD) and dementia with Lewy bodies (DLB), constitutes the second most common cause of neurodegenerative dementia after Alzheimer’s disease (AD). LBD is associated with significant disability, caregiver burden, and healthcare costs [1]. Despite its prevalence, studies indicate dementia in PD is underdiagnosed in clinical practice, [2, 3] and DLB is often misdiagnosed as Parkinson disease (PD) or Alzheimer’s disease, resulting in delayed diagnosis [1, 2, 4]. Underdiagnosis, delayed diagnosis, and misdiagnosis may prevent LBD patients from receiving appropriate care [5–7]. Therefore, identifying more reliable biomarkers for early and accurate diagnosis is a critical unmet need.

Cognitive fluctuations (CF) are a core clinical feature of LBD that are defined as spontaneous, transient changes in attention and alertness [8, 9]. These fluctuations are observed in up to 90% of patients with DLB [10, 11] and are associated with accelerated cognitive decline, increased caregiver stress, and reduced quality of life [10, 12]. Current recommended clinical assessments, such as the Clinician Assessment of Fluctuation (CAF) scale, rely heavily on patient and informant reporting and expert interpretation, which can introduce subjectivity and variability [13]. The 2017 DLB Consortium emphasized the need for objective, reproducible biomarkers of CF to improve diagnostic accuracy and facilitate early intervention [8, 14].

Electroencephalography (EEG) is non-invasive, accessible, and cost-effective, and it offers a promising biomarker for identifying CF. EEG can capture dynamic cortical activity with high temporal resolution, making it particularly suitable for detecting transient phenomena such as brief cognitive fluctuations. Based on a large body of work, quantitative EEG studies have revealed characteristic alterations in LBD, resulting in the inclusion of “prominent posterior slow-wave activity on EEG with periodic fluctuations in the pre-alpha/theta range” as a supportive biomarker for DLB [8]. A smaller number of studies have reported that CF in LBD was associated with lower dominant frequency and higher dominant frequency variability on EEG [15–19].

The objective of this study was to identify the combination of EEG features associated with CF across the Lewy body (LB) disease spectrum of DLB, PDD, and PD participants and to evaluate if EEG could be used as a biomarker of CF. Identifying EEG features linked to CF may not only enable the development of an objective biomarker for early and accurate diagnosis but also provide novel insights into the neurophysiological mechanisms underlying this poorly understood phenomenon.

## Methods

### Study design and participants

We conducted a cross-sectional, case-control study designed to identify EEG correlates of CF in LB disease. Patients were enrolled between 2021 and 2025 from Virginia Commonwealth University (VCU) Department of Neurology outpatient clinics. Eligible participants were 50–90 years old and had a clinical diagnosis of PD, PDD, or DLB according to standard criteria [8, 9, 20]. We excluded participants based on the following criteria: 1) history of another primary cognitive, psychiatric, or neurological disorder other than PDD or DLB; 2) deep-brain stimulator or any other neurosurgical history; 3) structural brain disease or known cerebrovascular disease; 4) history of seizures and/or use of a sodium-channel blocker; 5) regular use of benzodiazepines or barbiturates (or if taken as needed, then excluded if taken within five half-lives of study visit); and 6) severe dementia. Participants were required to have an informant if dementia was present. This study was approved by the Institutional Review Board at Virginia Commonwealth University (HM20020074). All participants or their legally authorized representatives provided written informed consent.

### Clinical Assessments

Clinical and neuropsychological assessments were completed during the same visit as EEG acquisition. The presence of CF was determined using the Clinician Assessment of Fluctuation (CAF) administered by an experienced clinician with an informant present. In those with the presence of fluctuating alertness and/or confusion, the frequency and duration of fluctuating confusion were each rated 0–4 and multiplied to yield a total score. For this study, any score greater than 0 was used to indicate the presence of CF. Participants also completed neuropsychological testing including the Montreal Cognitive Assessment (MoCA).

### EEG Acquisition

We asked patients to avoid caffeine, nicotine, and alcohol for at least 48 hours before their research visit. After the clinical evaluation, registered EEG technicians applied 19 silver-silver chloride scalp electrodes on patients lying in a low-lit, quiet, electrically shielded room using the International 10–20 System with standard reference/ground placement. Impedances were maintained <5kΩ. EEG was acquired using Natus XLTEK systems (Natus Medical Inc., Pleasanton, CA, USA) at 256 Hz, bandpass filtered from 1–70Hz, and notch filtered at 60Hz. EEG recordings used in this study were acquired in the eyes closed condition for at least 3 minutes. During EEG recording, participants were asked every minute if they were awake and were coached to reduce eye movements.

### EEG Processing and Analysis

EEGs were reviewed using Natus NeuroWorks 9 and Persyst 12 software to confirm awake background activity with minimal artifacts. Segments contaminated by eye movements, muscle activity, or drowsiness were excluded. For each participant at least 3 minutes of EEG was exported in EDF format. We processed EEG data using the EEGLAB toolbox (Swartz Center for Computational Neuroscience) in MATLAB R2024a (The MathWorks, Inc., Natick, Massachusetts, USA). We excluded artifacts using visual inspection of the independent component analysis (> 90% signal not derived from brain). We divided the remaining data into 90 two-second epochs. EEG data were band-pass filtered between 3 and 14 Hz prior to spectral feature extraction. EEG spectral features were extracted using a custom MATLAB-based Frequency Extraction (FE) toolbox. Power spectral density was estimated for each epoch using a fast Fourier transform (FFT) with a frequency resolution determined by the epoch length (2 s). Spectral analysis was performed on each epoch independently. The power spectrum was divided into four predefined frequency bands: delta (≤ 4.0 Hz), theta (4.5–5.5 Hz), pre-alpha (6.0–7.5 Hz), alpha (8.0–12.0 Hz), and beta (> 12.0 Hz) [21]. These band definitions were selected a priori and implemented identically across all participants. Dominant frequency (DF) was defined as the single frequency bin exhibiting the maximum spectral power within each epoch. Dominant frequency variability (DFV) was calculated as the standard deviation of DF values across all analyzed epochs for each channel. Dominant frequency prevalence (DFP) was defined as the percentage of epochs in which the DF fell within each predefined frequency band. EEG features were computed at the individual channel level and subsequently averaged within anatomically defined regions of interest: anterior (Fp1, Fp2, Fz, F3, F4, F7, F8), posterior (Pz, P3, P4, O1, O2), and temporal (T3, T4, T5, T6) derivations. The Frequency Extraction (FE) toolbox is publicly available for academic, non-commercial use at GitHub (https://github.com/Negidamd/negida-fe-toolbox) and archived on Zenodo [22].

### Statistical Analysis

Participants were categorized as LB disease with CF (LB + CF) or Lewy body disease without CF (LB-CF), based on the CAF. Demographic, clinical, and EEG feature data were aggregated, merged, and analyzed. Continuous variables were summarized as medians with interquartile range (IQR) and categorical variables were summarized as counts with percentages. Subject-level EEG metrics were averaged across channels within regions, and group summaries are reported as medians (IQR). Group differences were tested using Mann-Whitney U or Kruskal-Wallis tests for continuous variables and χ^2^ tests for categorical variables. To identify EEG predictors of CF, we performed logistic regression models adjusted for age, sex, and MoCA score. Highly correlated EEG features were reduced using elastic-net logistic regression (combining L1 and L2 penalties) to obtain a parsimonious multivariate model. Results were corrected for multiple comparisons by using a false discovery rate (FDR) threshold of *q* < 0.05. Analyses were performed in R v4.0.2.

## Results

### Characteristics of the study groups

A total of 53 participants met inclusion criteria and were included in the analysis: 29 with cognitive fluctuations (LB + CF) and 24 without (LB-CF). For the LB + CF group, the median CAF score was 8 [IQR 6–12]. Within the LB + CF group, there were 16 DLB, 11 PDD, and 2 PD participants, whereas the LB-CF group included 4 DLB, 4 PDD, and 16 PD participants. Median age was comparable between the LB-CF and LB + CF groups (73.5 vs. 73 years, respectively). Years of education were not significantly different between the two groups (LB + CF: 16 years vs. LB-CF: 18 years, P = 0.096). The LB + CF group included proportionally more males (79% vs 54%) and more DLB cases (55% vs 17%) than the LB-CF group. As expected, global cognition was lower in the LB + CF group (MoCA score = 20 [IQR 15–22]) compared to LB-CF group (24.5 [IQR 22–26]; p = 0.0015).

### Global and Regional EEG Characteristics

Compared with the LB-CF group, the LB + CF group demonstrated significant global and regional EEG slowing. DF and IAF were significantly lower across all scalp regions (all *q* < 0.005), and this slowing was accompanied by a shift in DFP from the alpha band to the pre-alpha and theta bands (Table 1).

Across all derivations, DFP alpha was markedly lower in the LB + CF group versus LB-CF (8.01% vs 50.09%; *p* < 0.001; *q* < 0.001; *r* = 0.59). DFP pre-alpha and DFP theta were correspondingly higher in LB + CF. The global DFP showed a shift from alpha to pre-alpha/theta activity in the LB + CF group. DFV showed a modest trend toward lower variability in LB + CF, particularly over anterior leads (frontal *q* = 0.012), but differences were small in magnitude. The largest effect sizes were observed for global DF (median 5.94 Hz vs 8.06 Hz; *p* < 0.001, *r* = 0.57) and IAF (8.02 Hz vs 8.55 Hz; *p* = 0.0002, *r* = 0.51).

### Relationship Between EEG Features and Cognitive Fluctuations

**Figure 1:**
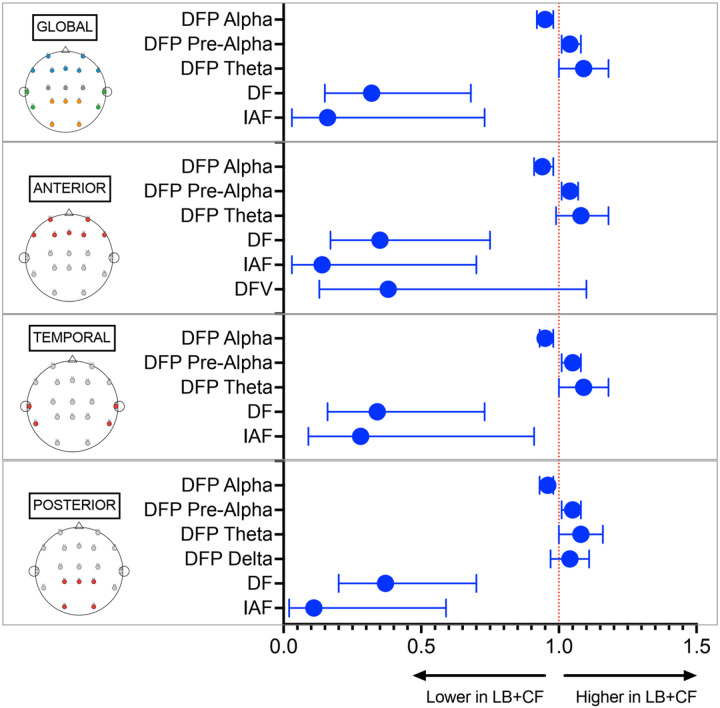
Forest plot showing the OR and the corresponding 95% CI for EEG features from logistic regression analyses adjusted for sex, age, and MoCA score. Abbreviations: DF = dominant frequency; DFP = dominant frequency prevalence; DFV = dominant frequency variability; IAF = individual alpha frequency; LB+CF = Lewy body dementia with cognitive fluctuations.

For EEG features that were significantly different between LB-CF and LB + CF, we performed logistic regression models adjusted for age, sex, and MoCA score. These demonstrated that features reflecting EEG slowing were significant independent predictors of CF (Fig. 1; Supplementary Table 1). The most predictive features were DF and IAF, both showing large effect sizes and consistent associations across cortical regions (global DF: OR 0.32, 95% CI 0.15–0.68, *p* = 0.003; global IAF: OR 0.16, 95% CI 0.03–0.73, *p* = 0.018). Reduced DFP alpha was also independently associated with CF, particularly in anterior and posterior regions (anterior DFP alpha: OR 0.94, 95% CI 0.90–0.98, *p* = 0.003; posterior DFP alpha: OR 0.96, 95% CI 0.93–0.98, p = 0.003). In contrast, DFP pre-alpha demonstrated a reciprocal positive association (OR 1.04, 95% CI 1.01–1.08, *p* = 0.011), consistent with a redistribution of spectral power toward slower frequencies.

### Machine learning predictive modeling

**Figure 2:**
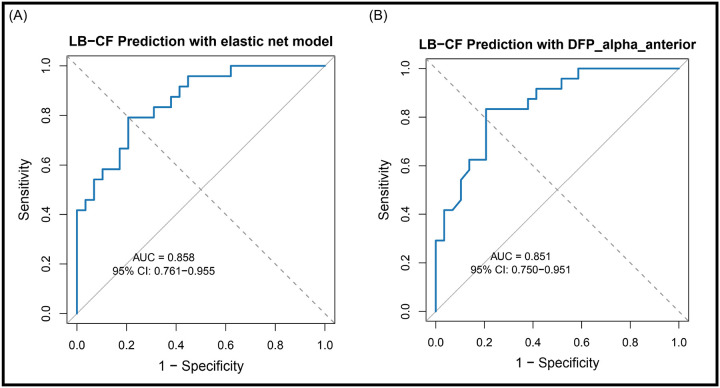
Receiver operating characteristic (ROC) curves comparing the five-feature elastic-net model (AUC = 0.858) and the single-feature model using anterior DFP-alpha alone (AUC = 0.85) for classification of LB+CF versus LB-CF. Abbreviations: AUC = area under the curve; DFP = dominant frequency prevalence; LB+CF = Lewy body dementia with cognitive fluctuations; LB-CF = Lewy body disease without cognitive fluctuations.

Starting with 28 candidate EEG features, we reduced dimensionality by removing highly correlated variables, yielding 10 features for model selection. An elastic-net logistic regression model identified six predictors, anterior DFP alpha, anterior DFP pre-alpha, anterior DF, temporal DF, temporal IAF, which together achieved an area under the ROC curve (AUC) of 0.86. When evaluated individually, each feature demonstrated more modest discrimination (AUC = 0.73–0.85). To test for parsimony, forward selection with AIC criteria identified a single feature, anterior DFP alpha, that achieved nearly equivalent performance (AUC = 0.85) compared with the full five-feature model. This finding indicates that anterior DFP alpha alone captures most of the discriminative power distinguishing LB disease patients with and without CF (Fig. 2).

## Discussion

### Summary of the key findings

In this study, we found that CF in LB disease was associated with a pattern of global EEG slowing characterized by reduced DF and IAF, substantially reduced DFP alpha, and reciprocal increases in DFP pre-alpha and theta. An elastic-net logistic regression model identified a six-feature EEG signature (AUC = 0.86) that detected the presence or absence of CF, but anterior DFP alpha alone yielded nearly identical performance (AUC = 0.85).

### Relationship to previous EEG work in LB disease

The general pattern of global EEG slowing that we observed is consistent with a large body of work showing that DLB and PDD display more pronounced slowing than AD and healthy controls, characterized by a shift of the dominant rhythm from the alpha range (8–13 Hz) into pre-alpha and theta bands [15, 16, 21, 23–25]. Bonanni and colleagues showed that early DLB and PDD are characterized by early pre-alpha DF and increased DFV, which discriminate LBD from AD and predict clinical progression [16, 21]. Van der Zande et al.[23] and Cromarty et al.[25] reviewed the literature and concluded that quantitative EEG measures, especially DF and posterior slow-wave activity, are promising biomarkers in LBD [8, 23, 25, 26].

EEG slowing is also evident in prodromal DLB (MCI-LB), correlates with core symptom severity, and differentiates MCI-LB from MCI-AD [17, 23, 27]. Stylianou et al. reported that DFV correlates with CF severity in the LBD population [15]. Our findings extend this work by showing that within a group of DLB, PDD, and PD patients, CF was associated with greater EEG slowing, beyond that seen in patients without CF. Similarly, Bonanni et al. demonstrated that the pre-alpha/theta DF characteristic of DLB was present early in the disease course, tracked with core clinical features including CF, and remained relatively stable over a two-year follow-up, suggesting a trait-like electrophysiological pattern [16]. Walker et al. established that DLB patients exhibit greater variability in slow electrocortical activity than AD patients [19], and Schumacher et al. showed that similar EEG slowing is already detectable at the MCI-LB stage [17].Taken together, these studies and our findings support a model in which progressive EEG slowing reflects the underlying neurophysiological substrate of CF across the full LB disease spectrum.

Our observation that DFV was modestly lower in the LB + CF group warrants comment, as earlier studies by Bonanni et al. and Walker et al. reported that CF severity correlated with *higher* DFV in DLB [16, 19]. However, several factors may explain this discrepancy. First, methodological differences in DFV calculation are substantial: Bonanni et al. used visual rating of DF range on sequential EEG segments, whereas we and others [15, 23] calculated DFV as the standard deviation of DF across epochs. Second, van der Zande et al. similarly reported lower peak frequency variability in pure DLB compared to AD (0.38 Hz vs 0.4 Hz, p < 0.05) [23], suggesting that our findings are not anomalous. Third, the inclusion of PD participants without dementia in our LB-CF comparator group may have increased apparent DFV in that group, as PD without dementia retains more alpha-range activity and thus greater potential for frequency fluctuation. Finally, when the dominant rhythm is restricted to a narrow low-frequency band, a floor effect may limit measurable variability, consistent with Schumacher et al.’s hypothesis that CF reflects reduced rather than increased variability in large-scale oscillatory activity and network interactions[24].

### Anterior versus posterior EEG slowing

Our results indicate that anterior DFP alpha was the single most informative feature for classifying CF. This is consistent with recent work by Franciotti et al., who re-analyzed multicenter DLB EEG data and reported that DF can be even slower in anterior than posterior derivations, and that this anterior slowing is associated with cognitive impairment and may reflect thalamocortical dysrhythmia [28]. Because anterior alpha is closely linked to frontoparietal attention networks and cholinergic/arousal systems, its reduction is a plausible correlate of CF. Given the high inter-correlations across regions (r = 0.94–0.98), anterior DFP alpha may reflect global slowing rather than region-specific physiology.

### Cognitive fluctuations and brain network dysfunction

**Figure 3:**
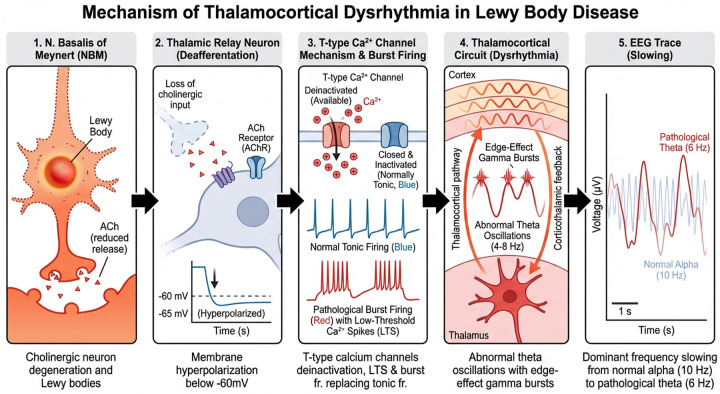
Proposed mechanistic pathway linking cholinergic pathology to EEG slowing in Lewy body disease. (1) Lewy body accumulation in the nucleus basalis of Meynert (NBM) causes degeneration of cholinergic projection neurons. (2) Loss of cholinergic input to thalamic relay neurons results in hyperpolarization and deinactivation of T-type calcium channels. (3) Subsequent low-threshold calcium spikes trigger burst firing, replacing normal tonic firing. (4) Burst-firing thalamic neurons entrain cortical networks into low-frequency theta oscillations, producing thalamocortical dysrhythmia. (5) Scalp EEG shows a shift from normal alpha rhythm (~10 Hz; blue) to pathological theta/pre-alpha rhythm (~6 Hz; red). This slowing correlates with cognitive fluctuation severity. Arrows indicate causal relationships; color coding distinguishes normal (blue) from pathological (red/orange) states.

CF are increasingly understood as manifestations of unstable large-scale brain network dynamics, with EEG, MEG, and fMRI studies linking CF to impaired thalamocortical and frontoparietal network integration [15, 24, 29–33].These network abnormalities have been mechanistically tied to degeneration of the cholinergic basal forebrain, particularly the nucleus basalis of Meynert [34–43]. Accordingly, the reduced DFP-alpha and lower DF/IAF observed in our LB + CF group likely reflect diminished cholinergic drive and impaired thalamocortical oscillatory stability (Fig. 3).

### Clinical implications

Our data have several implications for diagnosis and clinical research. The strong association between CF and reduced anterior DFP alpha suggests that simple EEG measures could provide a more objective, quantifiable correlate of CF. This is consistent with prior EEG work showing links between CF and DF [15] and with network-based studies tying CF severity to dynamic connectivity instability [29, 31–33, 44]. Our findings suggest that adding region-specific features such as reduced anterior DFP alpha may refine the supportive EEG biomarker listed in current DLB criteria. Our results could inform future research to identify individuals at high risk of developing CF through longitudinal studies in prodromal cohorts. Prior work showed that cholinesterase inhibitor benefit is more pronounced in patients with EEG slowing [45–50], suggesting DF and DFP alpha may serve as pharmacodynamic markers or stratification tools in trials.

### Strengths and limitations

Major strengths of this study include: (i) a prospectively enrolled LB disease cohort with standardized, clinician-rated CF assessment; (ii) the use of intuitive spectral features (DF, DFV, DFP in predefined bands) that are easily implemented with standard clinical EEG systems; and (iii) a combined classical and machine-learning analytic strategy that allowed us to both identify an interpretable biomarker (anterior DFP-alpha) and benchmark its performance against a multi-feature model.

However, several limitations warrant consideration. First, the cross-sectional design precludes conclusions about the temporal relationship between EEG changes and the emergence or evolution of CF. Longitudinal studies are needed to determine whether worsening EEG slowing predicts emergence of clinical CF. Second, the study was conducted at a single center, and external validation across different EEG systems and acquisition protocols will be essential to confirm generalizability. Finally, while we adjusted for age, sex, and global cognition, residual confounding by unmeasured factors (e.g., vascular burden and sleep quality) remains possible.

### Future directions

Future research should prioritize multicenter validation and harmonization of DF- and DFP-based EEG metrics to ensure reproducibility across recording systems, preprocessing pipelines, and clinical settings. Longitudinal and multimodal biomarker studies, integrating structural and functional MRI, cholinergic PET, and emerging α-synuclein biomarkers, are needed to clarify how EEG slowing relates to pathology, cholinergic degeneration, and moment-to-moment fluctuations. Finally, therapeutic and neuromodulation trials should evaluate whether EEG metrics, particularly DFP alpha and DF, could serve as treatment-responsive endpoints, stratification tools, or surrogate markers for cholinergic and network-targeted interventions in LBD.

## Conclusions

Overall, the present study adds to growing evidence that CF in patients with LB disease are accompanied by a shift from alpha to pre-alpha/theta DF on EEG, with reduced frontal alpha activity emerging as a particularly informative feature. Because these EEG metrics are relatively simple, low-cost, and scalable, they offer a practical biomarker for diagnosis and potential therapeutic monitoring in LB + CF.

## Supplementary Material

Supplementary Files

This is a list of supplementary files associated with this preprint. Click to download.

- SupplementaryTable1.docx

## Figures and Tables

**Table 1:** EEG features of Lewy body disease with and without cognitive fluctuations

	EEG Feature	LB + CF (n = 29)	LB − CF (n = 24)	p-value	FDR-adjusted p-value	Effect size (*r*)
Global	Frequency Prevalence					
	Alpha	8.01 (4.21–18.07)	50.09 (32.84–72.40)	<0.0001	0.0001	0.59
	Pre-Alpha	47.43 (23.57–54.80)	22.22 (6.99–42.20)	0.0053	0.0076	−0.38
	Theta	17.08 (5.73–26.02)	3.92 (2.10–8.35)	0.0005	0.0011	−0.48
	Delta	17.60 (10.53–28.54)	12.63 (6.24–18.84)	0.0473	0.0552	−0.27
	DF	5.94 (5.53–6.96)	8.06 (6.67–8.42)	<0.0001	0.0001	0.57
	IAF	8.02 (8.01–8.43)	8.55 (8.32–9.28)	0.0002	0.0005	0.51
	DFV	1.59 (1.35–1.88)	2.16 (1.59–2.49)	0.0435	0.0529	0.28
	DF Range	4.45–8.26	5.50–9.77			
Anterior	Frequency Prevalence					
	Alpha	7.14 (3.02–15.40)	45.88 (31.71–65.00)	<0.0001	0.0001	0.60
	Pre-Alpha	46.67 (22.38–53.97)	22.06 (8.29–43.77)	0.0045	0.0070	−0.39
	Theta	16.98 (5.71–22.70)	4.53 (2.37–7.70)	0.0008	0.0014	−0.46
	Delta	20.79 (11.59–33.97)	13.14 (8.05–25.48)	0.1002	0.1079	−0.23
	DF	5.77 (5.11–6.67)	7.18 (6.25–8.09)	<0.0001	0.0002	0.54
	IAF	8.02 (8.00–8.22)	8.48 (8.11–9.04)	0.0002	0.0005	0.51
	DFV	1.66 (1.46–2.17)	2.29 (1.76–2.72)	0.0091	0.0121	0.36
	DF Range	4.23–8.04	5.00–9.17			
Temporal	Frequency Prevalence						
	Alpha	7.22 (3.89–21.94)	59.02 (30.62–78.47)	<0.0001	0.0001	0.59
	Pre-Alpha	45.28 (25.56–56.11)	21.80 (5.76–37.98)	0.0032	0.0053	−0.40
	Theta	16.94 (5.83–25.00)	2.78 (1.60–8.33)	0.0004	0.0009	−0.49
	Delta	16.67 (8.89–29.44)	11.67 (5.41–17.43)	0.0619	0.0693	−0.26
	DF	6.15 (5.51–6.92)	7.90 (6.76–9.02)	<0.0001	0.0001	0.57
	IAF	8.01 (8.00–8.40)	8.52 (8.20–9.44)	0.0005	0.0011	0.48
	DFV	1.48 (1.34–1.70)	1.99 (1.30–2.40)	0.1478	0.1478	0.20
	DF Range	4.45–8.52	5.72–10.48			
Posterior	Frequency Prevalence					
	Alpha	6.44 (3.56–18.67)	58.34 (33.95–77.84)	<0.0001	0.0001	0.57
	Pre-Alpha	45.78 (25.78–56.89)	25.45 (4.56–41.11)	0.0055	0.0076	−0.38
	Theta	18.00 (6.89–24.89)	4.22 (1.56–9.56)	0.0006	0.0012	−0.47
	Delta	17.33 (9.56–24.89)	9.78 (4.05–13.66)	0.0155	0.0197	−0.33
	DF	6.01 (5.48–6.91)	8.36 (7.39–9.21)	<0.0001	0.0001	0.60
	IAF	8.01 (8.00–8.25)	8.73 (8.13–9.55)	<0.0001	0.0001	0.58
	DFV	1.56 (1.26–1.93)	1.74 (1.37–2.17)	0.1334	0.1383	0.21
	DF Range	4.18–8.47	4.88–10.56			

All values are reported as median (IQR) except for DF range. Mean DF, DFV, and IAF were calculated for each individual, and the group values are expressed as median Hz (IQR). A P < 0.05 indicates statistical significance after adjusting for multiple comparisons using FDR. Abbreviations: DF = Dominant Frequency; DFP = Dominant Frequency Prevalence; DFV = Dominant Frequency Variability; IAF = Individual Alpha Frequency; CF = Lewy Body Disease with Cognitive Fluctuations; LB-CF = Lewy Body Disease without Cognitive Fluctuations.

## Data Availability

Raw data of this study is available upon reasonable request to Matthew J. Barrett: matthew.barrett@vcuhealth.org
